# Nurse Midwives’ Perspectives on Women’s Vocalization During the First and Second Stages of Labor: A Qualitative Study

**DOI:** 10.3390/healthcare13192451

**Published:** 2025-09-27

**Authors:** Isabel Rute Pereira, Margarida Sim-Sim, Maria Otília Zangão

**Affiliations:** 1 Nursing Department, University of Évora, 7004-516 Évora, Portugal; rute.pereira@ipportalegre.pt; 2 Maternal Health and Obstetrics Department, Unidade Local de Saúde do Alto Alentejo, 7300-853 Portalegre, Portugal; 3 Health School, Polytechnic Institute of Portalegre, 7300-555 Portalegre, Portugal; 4 Comprehensive Health Research Centre (CHRC), São João de Deus School of Nursing, University of Évora, 7004-516 Évora, Portugal; msimsim@uevora.pt

**Keywords:** natural childbirth, humanized care, control, voice, nurse midwives

## Abstract

**Background/Objectives:** Women’s vocalization during labor can be highly influenced by the attitudes and practices of nurse midwives. This study analyzed the perspectives of nurse midwives regarding women’s vocalization in the first and second stages of labor (*n* = 16). **Methods:** Descriptive study using semi-structured interviews analyzed through lexical analysis with IraMuTeQ software. Data collection took place between February and April 2024. **Results:** The analysis revealed six thematic classes: “Attitude toward vocalization”, “Significance of women’s self-image”, “Resistance and acceptance of vocalization”, “Control and inhibition of vocalization”, “Advantages and disadvantages of vocalization”, and “Usefulness of vocalization in caregiving”. **Conclusions:** There are different attitudes among nurse midwives toward women’s vocalization, ranging from respect to paternalistic control. Practical implications include the urgent need for curricular modules on vocalization in midwifery training programs and the deconstruction of biases that silence women’s voices during childbirth.

## 1. Introduction

The profession of midwifery is considered one of the oldest in history, being referenced in various documents, such as ancient Hindu and Egyptian texts and in the Bible [1,2]. It is in an environment of safety and intimacy, of respect for physiology, for women’s rhythms, and for their instinctive needs that, with the care of midwives, childbirth has been taking place for centuries [1,3]. The behavior of women during natural childbirth followed the natural instinct and physiology inherent in the process [4]. It is in this context that women’s spontaneous vocalization is related to childbirth, given its physiological and instinctive nature [5,6]. Human vocalization precedes verbal production in evolutionary terms and encompasses a variety of sounds produced irregularly, mainly open and poorly articulated vowels, such as screams, cries, laughter, moans, among others [7,8,9]. 

The intensification of the medicalization of childbirth in the 20th century, despite its undeniable contribution to the drastic reduction in maternal and neonatal mortality and morbidity, led not only to the devaluation of the practice and ancestral wisdom of midwives, but also to the loss of women’s autonomy in their own childbirth [10]. Women in labor were expected to behave in a controlled manner, with discretion and obedience, fulfilling the role of good mothers. Consequently, the imposition of silence in the hospital environment repressed women from expressing themselves spontaneously, arousing feelings of shame and guilt for using their natural voice, which are still present today [11].

Currently, the trend toward humanization and demedicalization of childbirth focuses on women and their autonomy in a natural process [12], recognizing the importance and benefits of vocalization during labor [13,14,15,16]. In addition to valuing spontaneous vocalization as a nonverbal form of instinctive and natural communication during labor [5,6,17,18,19], the use of intentional vocalization is now part of a more conscious and empowering approach to women as active participants in the physiological process of childbirth [20]. Intentional vocalization involves learning and training with a specialist in the field and includes prenatal singing, vocal toning, theatrical vocal technique, and breathing technique with sound emission on exhalation, respecting the physiology of spontaneous vocalization [20,21,22,23].

Despite significant advances achieved to date in the humanization of childbirth, the weight of centuries of patriarchal society continues to impact the practices of healthcare professionals in birthing environments. In hospital settings, these influences manifest themselves through the domination and control of women’s bodies, acts of obstetric violence, and even social expectations about femininity and motherhood [24,25,26,27].

The philosophy of care of nurse midwives in hospital settings, although based on the obstetric care model [28], often incorporates principles from the biomedical model [29]. The tension between care models creates organizational barriers that restrict natural vocal expression during labor [18].

Despite recognition of the communicative and therapeutic value of vocalization, there is a significant gap between evidence-based understanding and clinical practice [18,20,22], which is one of the main reasons for conducting this study. Hospital environments and the attitudes of healthcare professionals can significantly affect women’s freedom to vocalize naturally [11,15,30,31,32,33].

The need to integrate humanized approaches into the care provided by nurse midwives to women in labor in the current context complements and justifies the relevance of the study. A review of the literature aims to contribute to the production of more knowledge on the subject [34].

Despite the terminology used in relation to the stages of labor, only the active phase of the first stage was considered in the data collection, which will be clarified in the methodology section. According to the WHO, the first stage of labor comprises cervical dilation up to 10 cm, followed by the second stage with fetal expulsion, also considering the division of the first stage into a latent phase, up to 5 cm, and an active phase from 5 cm of cervical dilation onwards [35].

This study aims to analyze nurse midwives’ perspectives on women’s vocalization during the first and second stages of labor. What is the nurse midwives’ perspective on the use of vocalization by women during the first and second stages of labor?

## 2. Materials and Methods

### 2.1. Study Design 

A descriptive and exploratory study was conducted using qualitative methodology within an interpretive/constructivist epistemological framework [36] to understand the experiences and interpretations of nurse midwives regarding women’s vocalization during the active phase of the first stage of labor and during the second stage of labor. The reason for choosing these particular stages of labor is based on the clear relationship between the expression of pain, namely through vocalization and contraction [17], taking into account that it is in the active phase of the first stage, in relation to the latent phase [37] and during the second stage, that the intensity of pain is highest [25].

Qualitative methodology is the best approach for this study, as it allows us to answer the initial research question [38].

The lexicometric approach using IraMuTeQ (version 0.8 alpha 7) and Descending Hierarchical Classification (DHC) was chosen over manual thematic analysis to provide descriptive robustness and reduce interpretative bias, while ensuring credibility, confirmability, and transferability through triangulation of researchers and transcripts validated by two authors.

### 2.2. Participants Recruitment

The sample consisted of nurse midwives from a public hospital, part of a Local Health Unit in Alto Alentejo, Portugal. Data collection was carried out individually for each nurse midwife in the postpartum ward, after providing care to a woman during the first two stages of labor, to obtain qualitative data.

Participants were selected by convenience sampling among nurse midwives working in a public hospital in Alto Alentejo, Portugal. The inclusion criteria were: nurse midwives who provided continuous and independent care to women during the first and second stages of labor, resulting in eutocic deliveries, starting in the active phase of the first stage or earlier, for Portuguese women aged ≥18 years, with low-risk pregnancies, without epidural analgesia, without oxytocin administration, and without vocal dysfunction. Exclusion criteria included complicated deliveries, medicated deliveries, and women who did not speak Portuguese.

The sample consisted of 16 nurse midwives (Table 1), defined when data saturation was achieved using the cut-off criterion strategy. Empirical signs of saturation included the absence of new semantic units in the last three interviews, with the homogeneity of the group making this threshold plausible, given their similar professional training and work context [39,40,41,42,43]. 

The interview guide was tested with two participants to ensure clarity and appropriateness.

Each nurse midwife was personally and individually invited to participate in the study and signed an informed consent form. The participation of the nurse midwives included in the study was determined based on their informed consent and availability. The interview was scheduled, by mutual agreement, for the day after delivery.

### 2.3. Data Collection and Analysis

Data collection took place between February and April 2024, in a private space, a nursing office of the Postpartum Service, without the presence of third parties, lasting approximately 20 min, on the day after delivery. Social desirability bias was considered because the interviews took place in the workplace, close to clinical practice. Conducting interviews in the workplace, particularly close to clinical practice, can exacerbate social desirability bias, leading respondents to adjust their answers to create a positive impression, avoid criticism, or gain social approval [44]. Creating a safe environment and ensuring confidentiality were the strategies used to reduce the pressure to respond honestly, improving the accuracy of the data obtained [45].

A semi-structured interview technique was used, based on a guide with open-ended questions [46], constructed based on the scientific evidence model supported by Kristen Swanson’s theory [47] (Appendix A).

The sociodemographic data collected were subsequently analyzed using the Statistical Package for the Social Sciences (SPSS) program, version SPSS Statistics 29.0 for Windows, using descriptive statistics [48].

The interviews were transcribed in full using the “Transcribe” feature in Microsoft 365 Office. Then, to ensure credibility and reliability, two of the authors validated the transcription. The corpus was formatted following the guidelines of the IraMuTeQ (Interface de R pour les Analyses Multidimensionnelles de Textes et de Questionnaires) software analysis protocol, version 0.8 alpha 7. The interviews (int_01 to int_16) were coded, as were the variables (years as a nurse midwife, age, gender, geographical origin, religion, marital status, academic qualifications). Thus, the 16 Initial Context Units (ICUs), corresponding to the 16 interviews that made up the analysis corpus, each began with a defined command line: **** *int_01 *ynm_1 *age_1 *gdr_1 geoo_1 *rlg_1 *ms_1*acadq_1. The Initial Context Units are transformed by the software into text segments with similar characteristics in terms of vocabulary, which are called Elementary Context Units (ECs), constituting the final classes.

The IraMuTeQ analysis processed 283 ECUs, with 262 classified (92.58% richness), generating 6 classes. Pre-processing included lemmatization, removal of irrelevant words, frequency limits (minimum of 3 occurrences), and chi^2^ significance (*p* < 0.001) for form retention. The non-classification rate was 7.42% (21 ECUs), indicating high analytical consistency.

The research team’s position as nurse midwives and educators potentially influenced data collection and interpretation. This was mitigated through research triangulation, systematic clustering of biases, and peer debriefing sessions to question interpretive assumptions.

### 2.4. Ethical Considerations

This study was submitted for prior approval to the Ethics Committee of the University of Évora (No. 22162), as well as for authorization to collect data, granted by the ethics committee of the institution where the study was conducted, ULSAALE (No. 05/2024, dated January 26). All necessary measures were taken to ensure the protection of the nurse midwives, namely anonymity and confidentiality. They received detailed information about the study, its purpose, and their contribution to scientific research. All nurse midwives gave their informed consent and were guaranteed anonymity.

## 3. Results

The results obtained, according to the defined data collection, are presented below.

### 3.1. Sociodemographic and Obstetric Characterization 

The sample consisted of nurse midwives aged between 38 and 61 years, with a mean age of 49.63 and a standard deviation of 8.57. The remaining characterization variables are presented in Table 2 below.

### 3.2. Semi-Structured Interviews 

**Figure 1:**
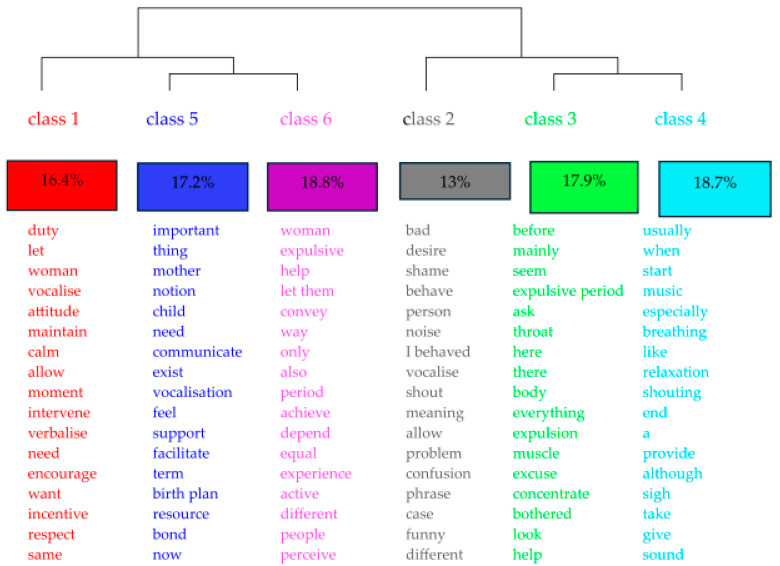
Dendrogram of the descending hierarchical classification (DHC) of interviews with nurse midwives.

The software classified 262 ECUs, resulting in six classes by descending hierarchical classification (DHC) (Figure 1). The processing of the interview data generated a textual corpus that was subsequently classified into thematic classes, revealing the representativeness of the content and the most representative words in each class, which were named as follows:

Class 1—Attitude toward vocalization

Class 2—Significance of women’s self-image

Class 3—Resistance and acceptance of vocalization

Class 4—Control and inhibition of vocalization

Class 5—Advantages and disadvantages of vocalization

Class 6—Usefulness of vocalization in caregiving

Figure 1 shows the formation of two classes, 1 and 2, which in turn gave rise to two classes each, classes 5 and 6 and classes 3 and 4. Appendix B complements and summarizes in a table the information relating to the class name, ECUs, %, representative words, and a brief illustrative quote.

Factor analysis shows that the intersection between classes 3 and 4 is representative of the relationship between resistance and acceptance of vocalization and its control and inhibition. In turn, the relationship between classes 5 and 6 reflects the link between the advantages and disadvantages of vocalization and its usefulness for caregiving. The themes of classes 1 and 2 are more distant, being, respectively, the attitude toward vocalization and the significance of women’s self-image. Class 1 gives rise to the advantages and disadvantages of vocalization (class 5), as well as its usefulness in caregiving (class 6). Class 2 gives rise to both resistance and acceptance of vocalization (class 3) and control and inhibition of vocalization (class 4). In terms of the percentages achieved by each class, class 4 was the most significant, representing 49 ECUs with a percentage value of 18.7%, followed by the closest class, class 3, containing 47 ECUs, with a percentage value of 17.9%. Next comes class 5, with 45 ECUs, representing 17.2%, and the closest class, class 6, with 44 ECUs, representing 16.8%. Finally, class 1 has a percentage value of 16.4% (43 ECUs) and, in last place, with the lowest representation, class 2 has 34 ECUs, representing 13%.

### 3.3. Thematic Classes

The six thematic categories, derived from the data analysis, are presented below:

Class 1—Attitude toward vocalization

Regarding the attitude of nurse midwives toward the vocalization of women in labor, analysis of the interviews revealed that this attitude can vary between respect and devaluation or even inhibition.

The results in this category show that nurse midwives respect nonverbal vocal expression and provide noninterventionist care, giving women autonomy in their decision to vocalize during labor.

“I don’t think we should intervene in that… we should allow it”

(int_03)

However, nurse midwives also adopt a more interventionist attitude in their interaction with women who vocalize, encouraging and guiding them to vocalize.

“I encourage women if they feel the need to express themselves vocally, I always encourage them”

(int_09)

The paternalistic view of care provided by nurse midwives manifests itself through practices of control and discouragement of vocalization by women in labor.

“I think it’s a waste of resources and energy on the part of the pregnant woman, who could be focusing her attention on breathing effectively to help minimize the pain of contractions.”

(int_06)

Class 2—Significance of women’s self-image

In this category, nurse midwives recognize that vocalization during labor can cause women to feel shame, guilt, and embarrassment, as they consider it to be socially inappropriate behavior.

“You still see some women who are ashamed to scream or draw attention to themselves, perhaps because they don’t want to behave badly”

(int_11)

The altered state of consciousness achieved by women during the expulsion phase is associated by nurse midwives with the innate and involuntary nature of vocalization.

“There are women who do not feel comfortable vocalizing, and when they do so involuntarily… they apologize… they feel… ashamed… during the expulsion phase… they do not feel well… they are in labor… when they come to their senses, they apologize… because they feel ashamed.”

(int_03)

The intervention of nurse midwives to break the stigma of vocalization, demystify its use, and restore the role of women in their own childbirth, breaking with the hospital practices of judgment and inhibition common among nurse midwives, is supported by the results obtained.

“It’s allowing them, telling them that it’s okay to scream as much as they want… allowing them to vocalize.”

(int_13)

Class 3—Resistance and acceptance of vocalization

Not all nurse midwives feel prepared to facilitate the use of vocalization by women, as it is not a common practice in a hospital setting.

“I’m not in the habit of saying ‘look, scream’ or ‘open your mouth’. I’m not ready for that yet, nor am I aware of it… to make the woman feel comfortable doing it, but maybe it’s more because I don’t know… I don’t remember that it can be a tool for her.”

(int_10)

There is also reference to the embarrassment of nurse midwives when the intensity of the sounds vocalized by women in labor interferes with the silence of the hospital environment, disturbing not only professionals but also other users. The years of experience of nurse midwives can shape or even mitigate the impact that this type of vocalization has on health professionals; however, the stigma surrounding women’s vocalization seems to remain present among health professionals.

“They really scream, scream, we know it bothers us, but I notice that it bothers me less. At the beginning of my practice, vocalization bothered me more than it does today.”

(int_02)

“There is no need to encourage this, otherwise maternity will soon seem like a screaming competition.”

(int_16)

The embarrassment about vocalization expressed by nurse midwives in their care seems to give way to acceptance and tolerance, especially when the woman is in the second stage of labor. Despite the accepted benefits throughout labor, it is in the second stage that vocalization comes to the fore, with a positive contribution to vaginal opening and fetal expulsion.

“I think vocalizing helps them, especially during the expulsion period… also in the active phase, because when they vocalize, it helps to open the vagina due to the way they vocalize, the pressure exerted on the diaphragm is also reflected in the vagina”

(int_03)

Class 4—Control and inhibition of vocalization

Class 4 consists of nurse midwives with a particular interventionist attitude, controlling or inhibiting vocalization. Their care is paternalistic in nature, guiding and directing the woman and her vocalization during labor in a controlling and domineering manner, discouraging screaming and instructing the woman to vocalize the letter “a.” The use of music, chosen by the nurse midwives, is also mentioned as a tool for labor, in which the midwife plays a leading role.

“Sometimes I even say, when I give the signal, start saying ‘a’ and sighing as if you want to let go, and they do that… I try to guide them in some way in this vocalization so that it is controlled and at a much lower tone, which is better for her, makes her relax, and is better for me”

(int_05)

Controlling vocalization during labor, particularly screaming, especially in the first stage, is still considered a necessary intervention by nurse midwives.

“Because she is disoriented and out of control, not in the active phase, I don’t encourage it. Sometimes I even tell women that if they want to scream, they should scream, but not in an uncontrolled way… when they are in the expulsion phase… if they want to scream, they should scream.”

(int_12)

The lack of recognition of the role of vocalization in labor, particularly in pain relief, leads nurse midwives to intervene in order to inhibit its use by women.

“There may be some extreme screaming, I don’t think that has ever happened, but if it does, you have to understand that it’s not because she’s screaming louder that it’s going to relieve her pain. You have to understand and try other means, such as relaxation, breathing, exercises, listening to music, but without exaggeration.”

(int_07)

The inhibition of vocalization and its replacement by silent breathing, considered more effective in the expulsive efforts of the second stage of labor, also emerges as a practice of nurse midwives.

“Breathing in and filling your chest with air and then breathing out slowly through your mouth while pushing is more effective because it is a sustained force, as the woman concentrates on that breathing and can hear the specialist nurse… in fact, it helped a lot, she didn’t scream, she breathed very effectively and pushed at the right moment, at the right time and for the right amount of time.”

(int_06)

Class 5—Advantages and disadvantages of vocalization

Nurse midwives recognize the importance of vocalization as a resource for women, with advantages for the fetus, facilitating and supporting the dynamics of labor, particularly in the second stage. They also recognize women’s lack of awareness of the advantages of vocalization, which is not addressed in the birth plan they draw up.

Regarding the advantages and disadvantages of vocalization in women in labor, most of the benefits identified were related to the use of vocalization by women in general during labor.

“I think it’s natural, and women used to do it in the old days, so maybe it really is beneficial for labor”

(int_04)

There is recognition of the important role in conveying information and expressing feelings of women who vocalize during labor.

“Vocalization is an important way for people to express what they are feeling”

(int_16)

The communication of pain through vocalization deserves special mention, relating it to personalized, woman-centered care.

“When we hear them vocalize, some expressions, we know if they are in pain, if they need something”

(int_08)

In addition to being a vehicle for transmitting information, vocalization may also play a role in relieving labor pains.

“If a woman is vocalizing, it is to relieve pain”

(int_09)

The continuity of mother-fetus and mother-baby communication through vocalization, whether during pregnancy, labor, or birth, is also highlighted.

“They hear their mother’s vocalization, they hear the voice they already heard in the womb… it is the continuation of the mother-child relationship that already existed”

(int_02)

Specifically in the expulsive period, vocalization is recognized as an advantage for effective expulsive efforts.

“Sometimes things are a little difficult, and they open their mouths and vocalize, and that seems to help a little, and they gain strength and energy to expel”

(int_10)

The relationship between vocalization and the exit of the fetal pole, facilitated by greater vaginal opening, is another of the advantages mentioned during the expulsive phase.

“The different forms of vocalization help to open the vagina”

(int_03)

On the other hand, vocalization, although related to the altered state of consciousness achieved by women in labor, can be a disadvantage, particularly in terms of the interference it can cause in the monitoring of fetal well-being by nurse midwives.

“Sometimes I think we need to bring them… back to reality, because sometimes we need them to do certain things to check the well-being of the fetus, but other than that… they can vocalize”

(int_04)

Class 6—Usefulness of women’s vocalization in caregiving

The recognition of the importance of vocalization as a useful tool for guiding caregiving is expressed by nurse midwives in their understanding and interpretation of women’s behavior throughout the first and second stages of labor, both in their experience and in their relationship with the fetus/newborn.

“We end up understanding better how patients feel and what their needs are”

(int_02)

“In the expulsion phase, it’s the same thing, because in the expulsion phase we can also understand a little about their reactions, how they view childbirth and not only that, but also birth, how receptive they are to receiving the newborn, their child”

(int_01)

Attentive listening and interpretation of women’s vocalizations during the first and second stages of labor are considered by nurse midwives as a guide for providing care.

“They don’t need to say words about what they are feeling; their expressions and what we hear are important so that we can perhaps know the best way to act in the active phase of the expulsion period. It’s the same thing.”

(int_08)

The information conveyed by vocalization can be quite diverse, ranging from requests for help, fear, pain, and expressions of well-being.

“Vocalization… conveys the moment… it can be a vocalization of well-being… and it can be distressing, asking for help… and we try to help”

(int_02)

“They also convey a little of what they are feeling in terms of pain and not only that; sometimes even fear, other times other needs they have in relation to the active phase”

(int_01)

## 4. Discussion

This is followed by a discussion of the results obtained through qualitative analysis of the interviews, organized according to the six thematic classes obtained. The discussion is supported by current scientific evidence that best supports each class.

Class 1—Attitude toward vocalization

The tension between respect and control emerged as a central theme, reflecting different philosophies of care and ways of dealing with women during labor. The contrast in the hospital environment between obstetric and technocratic models of care [49] is evident in attitudes toward the control of vocalization.

The obstetric care model favors minimizing interventions, supporting the natural process of childbirth in a respectful manner, including the woman’s vocal expression [50]. 

Respectful care in all aspects of labor is fundamental to a positive birth experience for all women and newborns and is recognized as a fundamental human right [51,52]. In vocalization or any other aspect of labor, ensuring respectful and dignified care has an impact on women’s well-being [53]. In this sense, clinical practices that avoid excessive medicalization [54] and respect physiology and natural vocalization contribute to improving healthcare and the satisfaction of women in labor [5,6]. Birth environments where nurse midwives provide calm, safety, autonomy, and intimacy encourage women to use their voices freely and without restrictions [18].

A more interventionist approach to care, whether encouraging and vocalizing simultaneously or verbalizing to discourage vocalization, has also proved to be a common practice, based on a technocratic model, in the context of labor. These interventions contribute to a loss of autonomy and empowerment for women [15,55]. Although there is currently a paradigm shift in obstetric care, which tends to focus on women’s autonomy, paternalistic and interventionist attitudes persist among health professionals, shaped by cultural, systemic, and historical factors [56,57]. The training of future nurse midwives can play a crucial role in implementing obstetric care models in maternity wards [58], naturally contributing to an attitude of respect and understanding towards the use of vocalization by women in labor [15].

Class 2—Significance of women’s self-image

Vocalization arouses feelings of shame, guilt, and embarrassment in women, reflecting behaviors of self-censorship. This observation highlights the weight and oppression that patriarchal society still exerts on women today [59].

The perpetuation of a culture of shame reinforces gender differences, resulting in higher levels of shame for women than for men [60]. It is from this perspective that women’s use of vocalization in various contexts, and during labor in a hospital setting, is marked by cultural prejudices rooted in history [20]. Behaviors that align with socially desirable norms for women, such as altruism, delicacy, kindness, and sympathy, clash with the free use of vocalization during labor, arousing feelings of shame [6,11,20].

Vocalization expressed through screaming, with its acoustic characteristics of high pitch and loud volume, is more associated by nurse midwives with embarrassment and shame in women in this study, perhaps due to the belief that it is categorized as an unacceptable or inappropriate sound, which causes confusion in a controlled environment [15]. However, nurse midwives recognize the inevitable nature of vocalization, explained by the altered state of consciousness achieved by women during natural childbirth [61,62], when they refer to allowing women to vocalize as they wish.

During the altered state of consciousness, also known as birth consciousness, women may experience a sense of control, loss of self-awareness, and a transformation of time. This state of consciousness is hypothetically considered an adaptive response to the pain and stress of childbirth, potentially improving the birthing process. The temporary reduction in prefrontal cortex activity that occurs during intense experiences such as childbirth establishes a bridge with the use of vocalization. Reduced self-awareness and altered perception of time may cause women to feel less inhibited in their vocal expressions [61,63]. 

The role of nurse midwives in deconstructing the stigma of shame associated with vocalization can be fundamental in removing barriers and creating conditions favorable to its use during labor [15]. Creating a welcoming and empathetic environment can contribute to this goal, providing comfort and encouraging uninhibited expression through vocalization [11]. Once again, the training of nurse midwives plays a fundamental role in enabling them to identify and support women in their altered state of consciousness during natural childbirth [64], as well as in pain management and emotional support [65].

Class 3—Resistance and acceptance of vocalization

Hospital quiet rules create environments where natural vocal expression becomes problematic, affecting both women’s self-image and professional comfort levels.

The lack of a practice that allows active listening to vocalization as a fundamental tool for personalized, comprehensive, and integrative care [5,6,17,18,19,21] by nurse midwives in this study reveals the need to address the gap in their training to deal with women’s vocalization [15]. 

A study exploring healthcare professionals’ perspectives on women’s use of vocalization during labor concludes that academic training in maternal health and obstetrics does not currently seem to include content that prepares future nurse midwives to provide care that integrates vocalization into their practice. The same study reveals that, among all professionals who work with women, only doulas receive training in this area [15].

The discomfort caused by vocalization in a hospital setting, as reported by nurse midwives, is mainly related to resistance to sounds that also cause embarrassment to women in labor, leading them to apologize for their behavior, i.e., high-pitched sounds such as screams [15]. The interpretation of these sounds as a maladaptive way of coping with labor by healthcare professionals, according to a study exploring the various sounds emitted by women in the second stage, may legitimize this conclusion [6]. However, there is no scientific evidence to prove a negative effect on the progression of labor due to these or other vocalizations [15].

Cultural and organizational issues, such as sound censorship, which exists in a controlled and ideally quiet environment such as a hospital, can also shed light on how healthcare professionals feel about a woman who vocalizes during labor [18,20,22]. Contributing to this assumption is the sexual symbolism associated with vocalization during childbirth, given its acoustic similarity to the sounds associated with sexual intercourse [6,18].

The acceptance of sounds by the nurse midwives in the study, even those with a high pitch, particularly during the expulsive phase of labor, is based on the belief that vocalization during fetal expulsion is beneficial and helps with vaginal opening by relaxing the throat muscles. Although there is no scientific evidence to prove this relationship, one study shows a link between the use of vocalization during the second stage of labor and a reduction in perineal lacerations greater than 2 cm [22].

Reviewing environmental standards in maternity wards, creating private environments, and using soundproof materials can be a practical way to help women in labor vocalize [66].

Class 4—Control and inhibition of vocalization

The controlling and paternalistic intervention of nurse midwives in the vocalization of women in labor is particularly evident in this class. This control manifested itself through an interventionist, often authoritarian practice based on the belief that greater relaxation is achieved through breathing, open vowel sounds such as the letter “a,” or sighs, rather than screams.

The belief in breath control during labor is not universally accepted in the scientific literature. Although some studies support the benefits of using breathing techniques to reduce pain and anxiety, as well as to increase maternal satisfaction and control, the results are often inconclusive or reveal little or no difference between the use of breathing techniques and their absence [67,68].

Paternalistic attitudes among healthcare professionals in obstetrics can involve making decisions on behalf of women, ignoring their autonomy and preferences, leading to experiences that are not very empowering [69,70].

The control of women’s vocalization during labor in a hospital setting can be understood in light of the paternalism exercised by health professionals, who, when intervening, limit or inhibit its use under the pretext of acting in the woman’s best interest [57].

The existing scientific evidence on the clinical practice of healthcare professionals who deal with women in labor is solid in defending vocalization support, whether spontaneous [5,6,18] or intentional [21]. A study with professional actresses reveals greater benefits of spontaneous vocalization, as a liberating experience, compared to intentional vocalization practiced during the prenatal period [19]. On the other hand, studies on intentional vocalization conclude that its use in conjunction with a partner or healthcare professional brings harmony and support to women during labor [21,23].

The creation of a safe environment by healthcare professionals, without reservations regarding vocal expression, allows women to fully experience childbirth [18]. On the other hand, the authoritarian behavior of nurse midwives who control and censor vocalization, silencing women in labor, can be considered within the spectrum of obstetric violence in its verbal and psychological forms [18,20,71], justifying a closer look at its presence in the hospital environment.

Addressing the vocalization of women in labor in the academic training of health professionals in the field of obstetrics could contribute positively to the creation of contexts favorable to its use, in a comprehensive, integrative, and humanized approach. The creation and implementation of training modules on spontaneous or intentional vocalization in obstetrics curricula, with practice in clinical vocalization listening and basic interpretation, is consistent with existing evidence on the clinical usefulness of vocalization for care guidance [6,72,73]. Training should also focus on programs that emphasize the importance of non-stigmatizing language and respectful communication [74]. The training of nurse midwives should not ignore the impact of their words and actions on the emotional and psychological well-being of women in labor [75].

Class 5—Advantages and disadvantages of vocalization

Nonverbal communication through vocalization has several functions: expressive (emotional release), regulatory (physiological coordination), and communicative (expression of needs).

In a natural childbirth setting, the expression of feelings and the expression of the experience of pain through vocalization emerge as a nonverbal form of communication [17,18].

The affective and physiological nature of vocalization is part of a set of instinctive behaviors experienced by women in an altered state of consciousness [9,61,62,76]. Communication between women and their fetuses during pregnancy and childbirth through vocalization improves the bonding process between mother and child, from the prenatal period to the postpartum period [23,77].

The pain relief achieved through vocalization is supported by studies that attribute analgesic properties to the sound and vibration produced [78,79,80]. The advantages related to the dynamics of labor, namely vaginal opening and fetal expulsion, are not corroborated by current scientific evidence. On the other hand, there are several advantages described in scientific evidence for women who use vocalization during labor, in addition to those mentioned by the nurse midwives in this study. In addition to the pain relief mentioned above, vocalization can lead women to states of greater relaxation and emotional balance [21].

The physiology inherent in vocalization during labor provides women with a more participatory experience of authenticity, autonomy, liberation, and empowerment [5,6,17,18,19,20,21,23]. Despite the existence of studies proving the potential of vocalization to improve both the woman’s experience and the care provided by healthcare professionals [5,6], vocalization is classified as disadvantageous when nurse midwives consider that it negatively interferes with their practice.

The results obtained in this class may convey a technocratic view of care provision by nurse midwives, insofar as the advantages of vocalization are recognized mainly for the labor process, with the advantages for women being secondary [81].

The practice of nurse midwives, although it tends to be based on the Obstetric Care Model, still has a risk-based approach today, influenced by the technocratic model, sometimes conflicting with recommendations and guidelines based on scientific evidence that combine skills with a physiological view of childbirth [49,82].

Class 6—The usefulness of vocalization in caregiving

Vocalization is recognized as a means of communication between women and the healthcare professionals who care for them, providing information about how they are coping with labor.

Listening carefully to vocalization can convey valuable information to healthcare professionals about a woman’s emotional, behavioral, and physical state [5,6,19,20]. The practical application of interpreting vocalization during moments of pain during labor is addressed in a study, which found that even nurse midwives with little experience can differentiate between the active phase and the expulsive period of labor [17].

The acoustic properties of women’s vocalizations during labor were also studied, making it possible to categorize behavioral states by associating them with different patterns of vocalization sounds [5,6]. Changes in vocalization during labor and their association with women’s physiological and emotional states are also documented in the “Voice and Effort Model,” which highlights the importance of the relationship between breathing, vocal production, and stress [83].

The development of skills in interpreting vocalization by healthcare professionals, applied to the provision of care, will lead to a practice that is less dependent on intervention and more respectful of physiology [5,6,17,18,21], in line with the current paradigm of humanizing childbirth [84].

Training in reading nonverbal communication as paralinguistic cues and expressive and regulatory functions in highly emotionally charged contexts, such as labor, which complement verbal communication, can also improve nurse midwives’ competence in interpreting women’s emotional states. Paralinguistic cues are indicators of this emotional state and are defined as tone, energy, or speed of speech. The expressive and regulatory functions of the voice are nonverbal expressions that convey emotion and help regulate speech flow in highly stressful situations, such as labor [83,85,86,87].

Concern about women’s silence, particularly during the second stage of labor, reinforces the negative impact that cultural and social aspects of gender shame have on freedom of expression [20,88,89,90].

Limitations of the study

Interviews conducted in the maternity ward, the day after delivery, and at participants’ workplaces may have inhibited criticism of service practices or emphasized socially acceptable responses.

Findings from this local hospital setting in the interior are not directly transferable to home births, birth centers, or different sociocultural contexts, emphasizing the need for culturally sensitive research approaches.

Although it provides descriptive robustness, this approach may attenuate interpretive nuances compared to manual thematic analysis, suggesting future triangulation studies.

## 5. Conclusions

This study provided information on nurse midwives’ perspectives on women’s vocalization during the first and second stages of labor, using a qualitative methodological approach based on interviews conducted after delivery.

This study revealed diverse attitudes among nurse midwives regarding women’s vocalization, reflecting broader tensions between humanized and technocratic models of care. The reformulated objective successfully captured the complexity of professional perspectives, identifying specific areas for improvement.

The main results reveal that nurse midwives have different attitudes toward vocalization, ranging from respect for natural expression to paternalistic practices characterized by control and inhibition. There is a clear recognition of vocalization as a natural, instinctive, and functional tool for communication and expression, integrated into the physiology of natural childbirth, in a respectful and woman-centered approach. However, paternalistic practices influenced by the technocratic model paradigm have also emerged, which clash with the principles of humanization of care, manifested by the control or inhibition of women’s vocalization.

It has also been found that vocalization, although a natural phenomenon, is also culturally mediated by beliefs and prejudices rooted in the patriarchal model of society, which materialize in the form of shame, guilt, and embarrassment, conditioning not only the experience of women but also the care provided by nurse midwives. Although the benefits of vocalization for both women and their care are recognized, the lack of formal training and the organizational context of the hospital environment, in addition to the cultural issues raised, converge in the devaluation of this aspect of childbirth by nurse midwives.

Specific recommendations include the following:

1. Incorporating curriculum modules on vocal physiology, toning techniques, prenatal singing, and clinical listening into obstetrics programs;
2. Standardizing environmental adjustments for acoustic privacy and advance information for the family;
3. Developing clinical language guidelines, replacing prescriptive directives with non-directive support techniques.

Future research should explore the effectiveness of the intervention, cross-cultural variations, and the long-term impacts of vocalization-based training on birth outcomes and satisfaction.

Recognizing and protecting vocalization as a physiological and communicative expression of women in labor is a fundamental pillar of the humanization of childbirth and, therefore, a right that services must guarantee.

## Figures and Tables

**Table 1:** Profile and codes of participants (*n* = 16).

Participant Code	Years as a Midwife	Age	Gender	Academic Qualification
int_01	19	57	Female	Postgraduate Diploma
int_02	24	55	Female	Master’s Degree
int_03	9	40	Female	Master’s Degree
int_04	10	40	Female	Postgraduate Diploma
int_05	18	49	Female	Master’s Degree
int_06	2	38	Male	Master’s Degree
int_07	18	56	Female	Postgraduate Diploma
int_08	18	56	Female	Postgraduate Diploma
int_09	1	42	Female	Master’s Degree
int_10	18	56	Female	Postgraduate Diploma
int_11	12	39	Male	Master’s Degree
int_12	17	61	Female	Postgraduate Diploma
int_13	16	52	Female	Master’s Degree
int_14	17	56	Male	Master’s Degree
int_15	6	38	Female	Master’s Degree
int_16	19	59	Female	Postgraduate Diploma

**Table 2:** Sociodemographic characteristics of participants (*n* = 16).

Variables	Categories	Frequencies n (%)
Years as a nurse midwife	0–5 years	2 (12.50%)
	6–10 years	4 (25.00%)
	11–15 years	1 (6.25%)
	16–20 years	9 (56.25%)
Gender	Female	13 (81.25%)
	Male	3 (18.75%)
Geographical origin	Iberia	16 (100%)
Religion	Christianity	15 (93.75%)
	Atheism	1 (6.25%)
Marital status	Married or civil union	16 (100%)
Academic qualifications	Postgraduate Diploma in Maternal Health Nursing and Obstetrics Nursing	7 (43.8%)
	Master’s Degree in Maternal Health and Obstetrics Nursing	9 (56.3%)

## Data Availability

Information about the data collection instruments will be made available upon request to the corresponding author. The data are not available due to ethical and privacy restrictions.

## Abbreviations

The following abbreviations are used in this manuscript:

DHC	Descending hierarchical classification
ECUs	Elementary context units
ICUs	Initial context units
IraMuTeQ	Interface de R pour les Analyses Multidimensionnelles de Textes et de Questionnaires
SPSS	Statistical Package for the Social Sciences
ULSAALE	Unidade Local de Saúde do Alto Alentejo
WHO	World Health Organization

## Appendix A

Semi-structured interview guide based on Swanson’s Theory:

− What do you think about the use of vocalization by women in active labor? (Class 6, Class 3)
− What do you think about the use of vocalization by women in labor during the expulsion phase? (Class 6, Class 3)
− Do you believe in vocalization as a skill for women in labor? (Class 5)
− What kind of support did you offer women who vocalized? (Class 1)
− Are you aware of the expectations of women in labor regarding the use of vocalization during the active and expulsion stages of labor? (Class2)
− What is your attitude toward women who use vocalization during the active stage of labor? (Class 3, Class 4))
− What is your attitude toward women who vocalize during the expulsion stage of labor? (Class 3, Class4)
− What is your attitude toward women who are unable to vocalize on their own during the active stage of labor? (Class1)
− What is your attitude toward women who are unable to vocalize on their own during the expulsion stage of labor? (Class1)
− In addition to your personal experience in assisting women in labor, are you familiar with any models or theories, or have you read any scientific articles on vocalization during labor? (Class1)
− What resources have you used to enable women in labor to vocalize? (Class 1)
− How do you encourage vocalization to create the bond between mother and child? (Class 5)

## Appendix B

**Table A1:** Summary table of classes.

Class Number	Class Name	ECUs	%	Representative Words	Illustrative Quote
1	Attitude toward vocalization	43	16.4%	duty, leave, woman, vocalize, attitude, maintain, calm, allow, moment, intervene, verbalize, need, encourage, want, incentive, respect, equal	“My attitude is somewhat expectant, letting things happen, always trying to intervene as little as possible” (int_11)
2	Significance of women’s self-image	34	13%	bad, desire, shame, behave, person, noise, I behaved, vocalize, shout, meaning, allow, problem, confusion, sentence, case, funny, different	“They feel ashamed… they think that screaming or moaning… is bad behavior, and then they apologize… they feel guilty” (int_01)
3	Resistance and acceptance of vocalization	47	17.9%	before, mainly, seem, expulsive period, ask, throat, here, there, body, everything, expulsion, muscle, excuse, concentrate, bothered, look, help	“During the expulsion period… this shout helps with the expulsion… only at the moment of expulsion… shouting before doesn’t help much” (int_12)
4	Control and inhibition of vocalization	49	18.7%	normally, when, begin, music, especially, breathe, as, relaxation, shout, finish, one, provide, although, sigh, take, give, sound	“I also love deliver babies to the sound of music. When there is no soundtrack, I usually turn on the radio, and when they start to lose control, I say, ‘Look, get into the rhythm of the music’ (int_05).
5	Advantages and disadvantages of vocalization	45	17.2%	important, thing, mother, notion, child, need, communicate, exist, vocalization, feel, support, facilitate, term, birth plan, resource, bond, now	“Vocalization is an important way for people to express what they are feeling” (int_16)
6	The usefulness of vocalization in caregiving	44	16.8%	woman, expulsive, help, let them, convey, way, just, also, period, achieve, depend, equal, experience, active, different, people, perceive	“Vocalization… also helps nurse midwives interpret how the woman is experiencing labor” (int_02)
